# Experimentally induced social threat increases paranoid thinking

**DOI:** 10.1098/rsos.180569

**Published:** 2018-08-01

**Authors:** Vanessa Saalfeld, Zeina Ramadan, Vaughan Bell, Nichola J. Raihani

**Affiliations:** 1 Department of Experimental Psychology, University College London, 26 Bedford Way, London WC1H 0AP, UK; 2 Division of Psychiatry, University College London, London, UK; 3 South London and Maudsley NHS Foundation Trust, London, UK

**Keywords:** paranoia, social rank, group affiliation, social threat, game theory

## Abstract

The ability to attribute intentions to others is a hallmark of human social cognition but is altered in paranoia. Paranoia is the most common positive symptom of psychosis but is also present to varying degrees in the general population. Epidemiological models suggest that psychosis risk is associated with low social rank and minority status, but the causal effects of status and group affiliation on paranoid thinking remain unclear. We examined whether relative social status and perceived group affiliation, respectively, affect live paranoid thinking using two large-*N* (*N* = 2030), pre-registered experiments. Interacting with someone from a higher social rank or a political out-group led to an increase in paranoid attributions of harmful intent for ambiguous actions. Pre-existing paranoia predicted a general increase in harmful intent attribution, but there was no interaction with either type of social threat: highly paranoid people showed the same magnitude of increase as non-paranoid people, although from a higher baseline. We conclude social threat in the form of low social status and out-group status affects paranoid attributions, but ongoing paranoia represents a lowered threshold for detecting social threat rather than an impaired reactivity to it.

## 1. Introduction

Social threat detection mechanisms should be sensitive to the harms that can be imposed by conspecifics, including aggressive attacks, ostracism, social exclusion, reputation damage and other forms of harm that have potential negative consequences for reproductive success and/or survival. Nevertheless, humans show striking variation in the tendency to attribute harmful intent to others and this is present as a continuum in the population, varying in accuracy and ranging in content from increased socio-evaluative concerns to frank paranoid delusions [1–3]. Here, we distinguish between paranoid attributions (the attribution of harmful intent, which may or may not be accurate), paranoid ideation (the extent of paranoid thinking, which may include accurate attributions of intent but which is known to increasingly correlate with over-estimate of social threat as intensity increases [4]) and paranoid delusions, where delusions have a clear persecutory theme. Clearly, paranoia is an important social and clinical concern, although the causal factors underpinning variation in paranoid ideation are still not fully understood.

Both genetic and environmental components contribute to paranoia [5,6]. Research on environmental predictors has consistently implicated adverse social conditions in promoting paranoid thinking, with those at the bottom of the social ladder being most affected. Paranoia is more common in people with low socioeconomic status, including those who live in poverty [7,8]. Social adversity also manifests in terms of discrimination and exclusion, and people from minority ethnic backgrounds report higher levels of paranoia [9], an effect which is ameliorated as ethnic density increases [10]. Additionally, perceived discrimination is associated with paranoia in ‘ultra-high risk for psychosis' individuals [11] and mediates the association between deprivation and paranoia [12]. Increasing evidence also suggests that the subjective perception of one's relative status in the world is also an important predictor of paranoia. For example, a lower perceived social rank compared to others predicts paranoid ideation [13] and psychosis-proneness [14]. In addition, low subjective social rank mediates the effect of childhood deprivation on later paranoid thinking [15].

Collectively, these findings suggest that interacting with partners who have a higher social rank or are from a social out-group will increase concerns about personal harm and these effects will be exaggerated among people who have higher levels of pre-existing paranoia. However, results from epidemiological studies are correlative and cannot establish causal effects. Despite the obvious need for experimental work in this area, very few (if any) studies have addressed the causal factors that drive paranoia in genuine social interactions with others. Freeman *et al*. [16] used a virtual reality task to induce negative social comparisons by manipulating height—a common marker for social rank [17]—leading to increased paranoia when participants interacted with taller virtual avatars. However, a more recent study where participants were asked to compare themselves with profiles of high and low status people found a clear impact on perceived social rank but no effect on paranoid ideation [18]. Although experimentally innovative, both studies used simulated social situations, potentially limiting their ability to engage the most relevant social cognitive processes that help us manage competition and cooperation with others in genuine social interactions [19,20].

Paradigms from game theory can be used to examine social decision-making and perception in genuine social interactions [21], and these approaches are now increasingly being used in psychiatry research [22–26]. For example, Raihani & Bell [26,27] have used the Dictator Game to explore how pre-existing paranoia affects social cognition and behaviour. The Dictator Game is a two-player game, where one individual (the ‘dictator’) decides how to split an endowment of real money that the partner (the ‘receiver’) must accept [28]. This task is useful for identifying paranoid attributions because the motives underpinning dictator decisions in the task are ambiguous: selfish dictators might be motivated to earn more money for themselves (self-interest) or by a desire to deny the partner of any money (harmful intent). This paradigm allows a measure of the tendency to infer harmful intent during a social interaction in which the participant is directly involved.

The Dictator Game also lends itself to testing the effects of social threat on paranoid attributions. We do this here, using two pre-registered studies asking whether (i) interacting with people from similar or differing social ranks and (ii) interacting with in-group or out-group individuals, respectively, affect paranoid attributions. Previous work indicates that paranoia positively predicts harmful intent attribution but not attribution of self-interest in this game [26]. We expected to replicate this effect, and additionally to show that attribution of harmful intent is greater in socially threatening scenarios, specifically when interacting with someone of a higher social rank and when interacting with an out-group partner (respectively). We also expected that pre-existing paranoia would exacerbate the response to social threat, such that highly paranoid people would show dysregulated responses to social threat. In other words, we anticipated we would detect an interaction between paranoia and experimental social threat in the strength of harmful intent attributions.

## 2. Methods

### 2.1. Participants

Data were collected in June 2017. This project was approved by the UCL Ethics board (project 3720/001). Participation was voluntary and informed consent was obtained. We recruited 2030 (1175 female, 851 male, 4 unknown; age: range 18–98, mean: 35.9 ± 0.27) US-based participants via Amazon Mechanical Turk (MTurk, http://www.mturk.com).

### 2.2. Procedure

*Part A.* Participants were paid $0.50 for *Part A*. Initially, participants completed the Green *et al*. Paranoid Thought Scales (GPTS) [29], a 32-item scale that captures pre-existing paranoid ideation across the full clinical and non-clinical spectrum. Then, participants (i) rated their subjective social status using the MacArthur Scale of Subjective Social Status [30] and (ii) rated their political affiliation on a slider scale of 0–100 (initialized at 50) where 0 = liberal and 100 = conservative (see electronic supplementary material for materials). The MacArthur Scale provides a valid and reliable rating of subjective social status that shows the expected relationship to objective social status indicators [31] and health outcomes [32]. Subjective ratings of political affiliation are externally valid and strongly predict group identification and voting behaviour [33]. Moreover, political affiliation is a particularly salient type of group affiliation and is associated with differences in identity, social norms and values [34] and predicts divergent in- and out-group preferences and inter-group behaviours in experimental studies [35].

*Part B.* We allowed a minimum interval of 10 days before recalling participants to take part in one of the two experimental tasks (hereafter, the ‘social status task’ and the ‘political affiliation task’, respectively). Inevitably, we experienced some attrition with this method and not all of the participants from *Part A* were re-recruited. Of the 2030 participants recruited to *Part A*, we successfully re-recruited 1242 to the social status task and 1308 to the political affiliation task. In each task, participants were paid a show-up fee of $0.20 and could earn $0.25–$0.75 depending on the decisions made by them and by their partner. All participants from *Part A* were invited to take part in both experimental tasks in *Part B* (minimum interval of 9 days between tasks, task order counter-balanced).

In both tasks, participants were cast in the role of receiver in a Dictator Game. In the social status task, participants were reminded that they had previously indicated their subjective social status on the MacArthur Scale, and were then allocated to one of the three conditions where they were informed that the dictator was of either (i) higher, (ii) lower or (iii) equal social status to themselves. In the political affiliation task, participants were reminded that they had indicated their political affiliation on a slider scale and were then allocated to one of the two conditions, where they were informed that the dictator had either the same or different political affiliation to them (hereafter in-group and out-group condition). We note here a slight discrepancy in the methods across these two tasks in that the participants were explicitly told that their partner knew the political affiliation of both players in the political affiliation task, whereas (due to an oversight) this explicit information was not provided in the social status task (in the latter, participants knew that status of the partner but were not explicitly told that the partner also knew the participant's own status). In both tasks, once participants received the information about their partners, the Dictator Game proceeded.

Dictators were endowed with $0.50 and could make a fair decision (send $0.25 to participant) or an unfair decision (send $0.00 to participant). Participants saw the dictator's decision and then rated on two separate slider scales (0–100, initialized at 50) the extent to which they believed the dictator's decision was motivated by the dictator's ‘desire to earn money’ (hereafter ‘self-interest’) and by the dictator's ‘desire to reduce your bonus' (hereafter ‘harmful intent’).

All participants answered three comprehension questions in each task. Participants that failed a multiple-choice comprehension question were given a second attempt to answer correctly (using a free-form answer to prevent guessing). Participants who still answered incorrectly (54/1242, 4.0%, in the social status task and 50/1306, 3.8%, in the political affiliation task) could participate but we included incomprehension as a variable in analyses.

*Part C.* Finally, participants were informed that they would interact in a task with a new partner but that this time they were the dictator and their partner was the receiver. These decisions are not presented or analysed: they were collected only so that we could truthfully inform participants that the dictator decisions they saw had indeed been made by real players in the task.

### 2.3. Pre-registered predictions

Predictions for each task were pre-registered separately (social status task: https://aspredicted.org/kp3y4.pdf; political affiliation task: https://aspredicted.org/zd9sr.pdf). Analyses conform to the predictions specified in these documents unless stated otherwise. One main deviation from all analyses that was omitted from the pre-registered documents was to include ‘task comprehension’ as a variable affecting participants' ratings of the dictator's intentions in both tasks. We include task comprehension in all models, reporting the effects of this variable and also checking that results are qualitatively robust to the exclusion of failed comprehenders.

### 2.4. Pre-existing paranoia predictions

First, we analysed the variables affecting the GPTS paranoia score. We predicted that social status would be negatively associated with paranoia, and we pre-registered an exploratory analysis of whether political affiliation had any effect on paranoia. Visual inspection of the relationship between social status and paranoia revealed a bimodal distribution, with peaks at either extreme end of the status distribution. To explore whether extreme social status values affected paranoia, we therefore ran an unregistered analysis, where we included two binary dummy variables: ‘High Status’ (=1 if individual scored 9 or 10 on the social ladder; 0 otherwise) and ‘Low Status’ (=1 if individual scored 1 or 2 on the ladder; 0 otherwise). As paranoia scores were extremely right-skewed, we converted paranoia score into a nine-level ordinal categorical variable [33], where each level of the factor had a minimum of 200 data points (mean = 225). We note that we omitted to explicitly mention this transformation in the AsPredicted documents, but we did state that we would explore the variables affecting variation in paranoia score using a cumulative link model, which by definition implies an ordinal categorical response term. This transformation was anticipated and pre-registered. The variables affecting pre-existing paranoia were explored using a cumulative link model (clm) [36], which allows an ordinal categorical variable to be specified as the dependent variable in a linear model.

### 2.5. Social status task predictions

We predicted that participants would make stronger harmful intent attributions when paired with higher status partners and with unfair partners. We expected an independent positive effect of paranoia on harmful intent attributions, and we expected paranoia to interact with (i) relative status and (ii) dictator fairness to produce exaggerated harmful intent attributions (see electronic supplementary material for all pre-registered predictions). Relative status was a three-level categorical variable describing the dictator's status relative to the participant's. We recoded this into two binary dummy variables (‘Higher Status’ and ‘Lower Status’, respectively). The effects associated with the terms ‘Higher Status’ and ‘Lower Status’ can, therefore, be understood as relative to the base category of ‘Equal Status’.

We ran two clms, specifying (i) ‘harmful intent attribution’ and (ii) ‘self-interest attribution’ as five-level ordinal categorical response terms. In each model, we included the following explanatory terms: ‘Age’, ‘Gender’, ‘Fairness’ (unfair/fair dictator), ‘Subjective Social Status', ‘Paranoia’, ‘Comprehension’ (0, all questions correct; 1, at least one incorrect), ‘Higher Status’ (0, dictator not higher status; 1, dictator higher status), ‘Lower Status’ (0, dictator not lower status; 1, dictator lower status). We also included all pre-registered two-way and three-way interactions (see electronic supplementary material).

### 2.6. Political affiliation task predictions

We expected that participants would infer greater harmful intent when paired with an out-group dictator, compared to when playing with an in-group dictator. As above, we also expected this effect to be most pronounced for more paranoid subjects. As above, we ran two clms with the following dependent variables: (i) harmful-intent attribution and (ii) self-interest attribution. We included the following explanatory terms in both models: ‘Age’, ‘Fairness’, ‘Gender’, ‘Comprehension’, ‘Paranoia’, ‘Partner’ (in-group/out-group), ‘Political affiliation’ (subject's own political affiliation). We also included all pre-registered two-way and three-way interactions (see electronic supplementary material).

### 2.7. Statistical approach

Data were analysed using multi-model selection with model averaging (described in [27,37,38] and in detail in the electronic supplementary material). Briefly, this approach involves specifying a global model (with all predictors and interactions of hypothetical importance) and then comparing all derivative submodels to determine which model, or set of models, is most consistent with the data. Models are evaluated on the basis of an Akaike information criterion, corrected for small sample sizes (AICc), with lower AICc values indicating a better model fit. The best model is the model with the lowest AICc value—but models that are within two AICc units of the best model are also likely to be consistent with the data. To account for the intrinsic uncertainty over which model is the true ‘best’ model, parameter estimates and confidence intervals are therefore averaged over the full top model set (using package MuMIn [39]). In our models, continuous input variables were standardized [40] and binary input variables were centred, so estimates can be considered on the same scale. All data and code are available at https://osf.io/bzhx3/.

## 3. Results

The mean subjective social status score was 5.01 ± 0.04 (range: 0–10). The mean political affiliation score was 41.8 ± 0.67 (range: 0–100), indicating a slight liberal bias. Of the 2027 participants who provided a political affiliation score, 104 (5.13%) gave a score of exactly 50, whereas 1142 (56.3%) scored less than 50 (i.e. liberal) and 781 (38.5%) scored greater than 50 (i.e. conservative). Paranoia scores ranged from 32 to 160 (mean: 54.8 ± 0.57), with 146 (7%) participants scoring above the clinical mean of 101.9 reported in [29].

### 3.1. Variables affecting paranoia score

**Figure 1.**
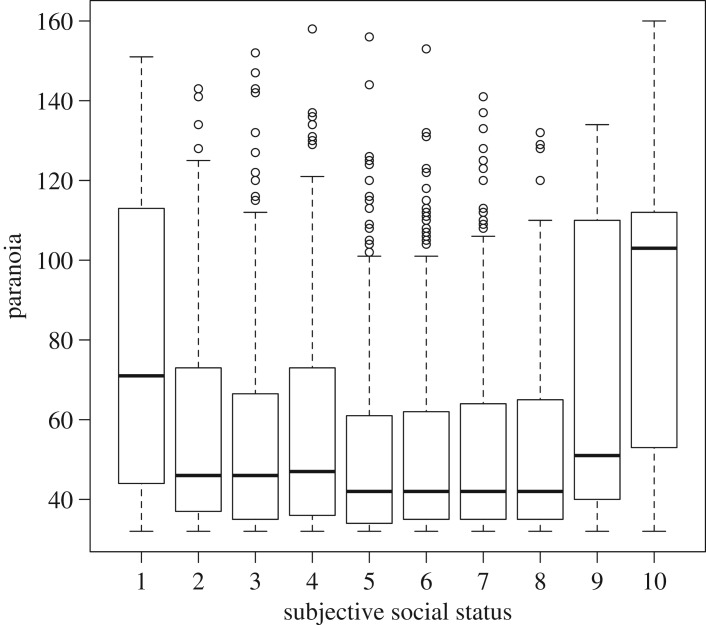
Boxplot and whiskers of pre-existing paranoia scores as a function of subjective social status. Boxplots display the median and the interquartile range; whiskers are minimum and maximum values that are less than 1.5× interquartile range. Circles are outliers.

**Figure 2.**
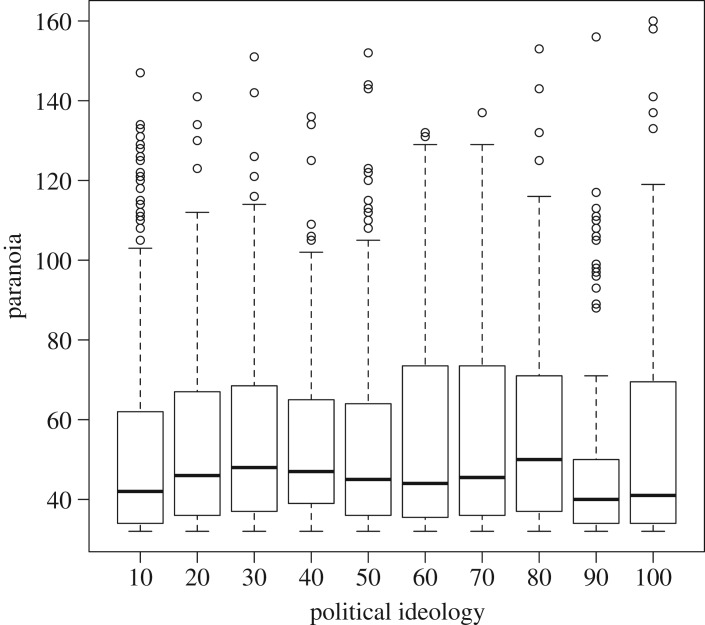
Boxplot and whiskers of pre-existing paranoia scores as a function of political affiliation (higher scores, increasing conservatism).

As predicted, social status had a negative effect on paranoia (estimate: −0.26, CI: −0.45, −0.06; figure 1*a*). However, participants who reported themselves as being on either rung 9 or 10 of the social ladder were also more paranoid than those below them (estimate: 1.31, CI: 0.67, 1.95; electronic supplementary material, table S1), even after controlling for the generally negative effect of social status on pre-existing paranoia (figure 1). Older participants were less paranoid (electronic supplementary material, table S1), while political conservatives were slightly more paranoid than political liberals (figure 2). We did not detect any meaningful effect of gender on paranoia (electronic supplementary material, table S1).

### 3.2. Harmful intent attribution

**Figure 3.**
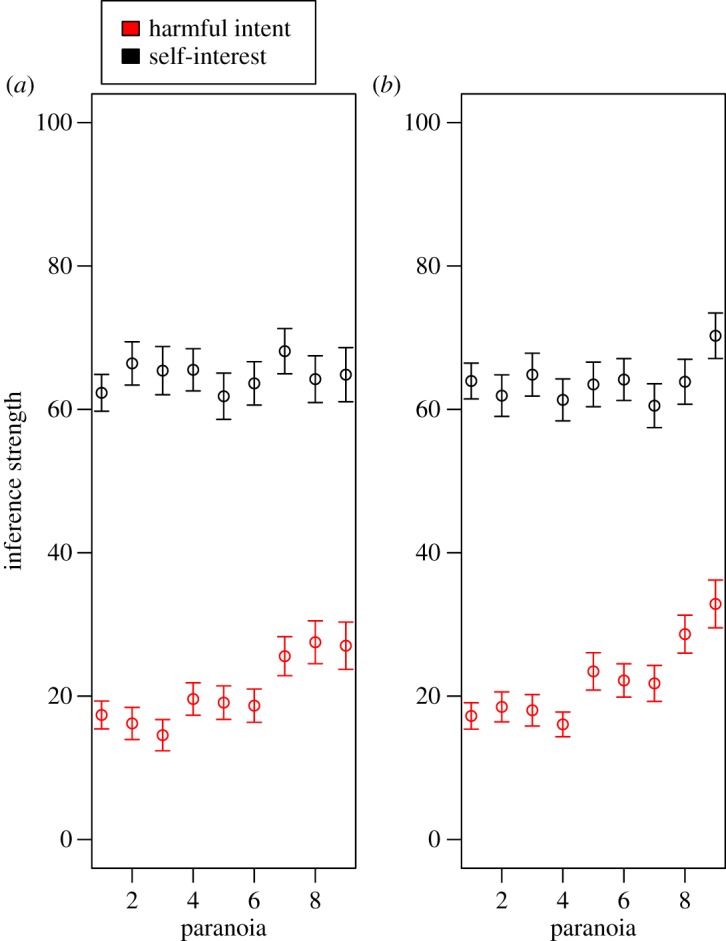
Mean harmful intent and self-interest attributions made by participants in the (*a*) social status and (*b*) political affiliation tasks, respectively. Means and standard errors are generated from raw data. For the ease of visualization, paranoia was converted to a nine-level categorical variable, though note that paranoia was included as a continuous term in the models.

**Figure 4.**
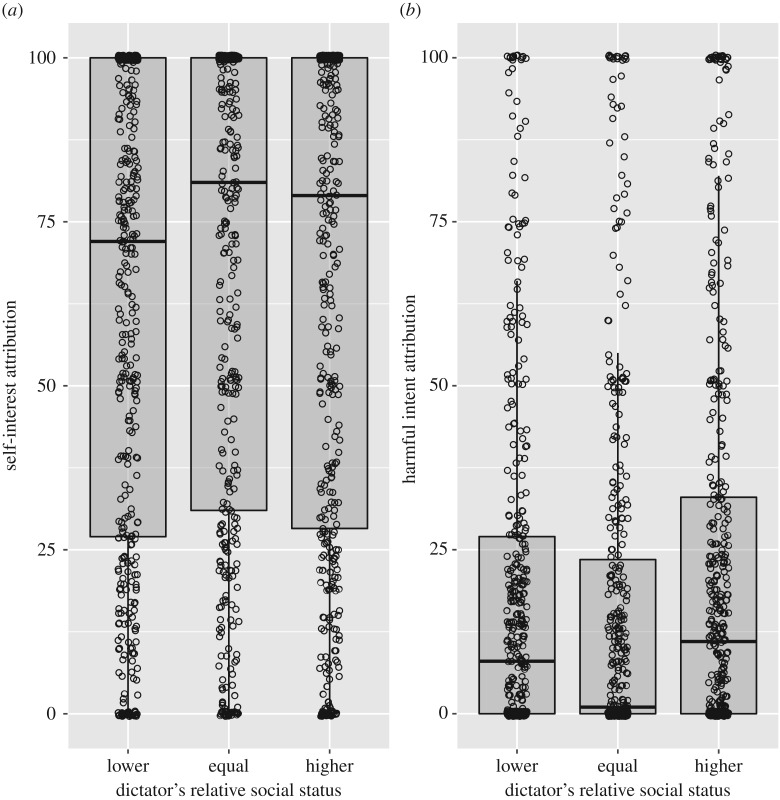
Boxplots of (*a*) self-interest attributions and (*b*) harmful intent attributions (generated from raw data) made for the dictator as a function of the dictator's relative social status. Raw data displayed as open circles, with a horizontal jitter function of 0.1 applied to ease visualization. Plot produced using R packages *ggplot2* [41] and *ggpubr* [42].

**Figure 5.**
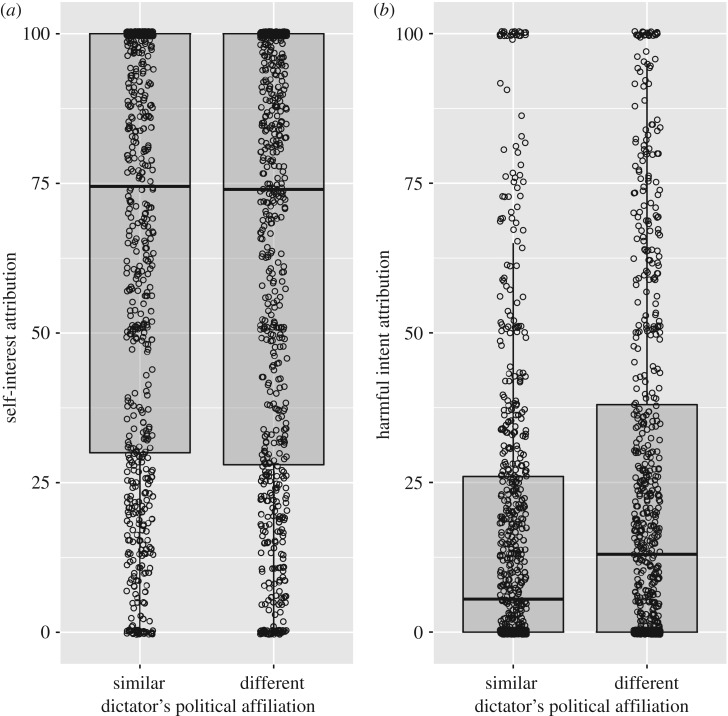
Boxplots of (*a*) self-interest attributions and (*b*) harmful intent attributions (generated from raw data) made for dictators who either had similar or different political affiliation to the participant. Plot produced with *ggplot2* [41] and *ggpubr* [42].

As predicted, harmful intent attributions were stronger when participants played against an unfair partner, both in the social status task (table 1) and in the political affiliation task (table 2). Paranoia positively predicted harmful intent attribution in both the social status task and the political affiliation task (tables 1 and 2; figure 3); and participants attributed more harmful intent to higher status partners (table 1; figure 4), and to out-group partners (table 2; figure 5), as predicted. Counter to our predictions, however, there was no interaction between paranoia and either relative social status or group affiliation. Thus, paranoid people did not show disproportionately strong harmful intent attributions when playing against higher status, or out-group, dictators and instead showed harmful intent attributions that were higher overall but nevertheless scaled up in response to social threat in the same way as for less paranoid participants (tables 1 and 2).

**Table 1.** Variables affecting harmful intent attribution in the social status task. Harmful intent was coded as a five-level ordinal categorical variable and set as the response term in a clm [36]. Importance is the probability that the term in question is a component of the true best model.

parameter	estimate	unconditional s.e.	confidence interval	relative importance
intercept 1|2	0.85	0.07	(0.71, 0.97)	
intercept 2|3	1.59	0.08	(1.44, 1.74)	
intercept 3|4	2.22	0.10	(2.03, 2.40)	
intercept 4|5	2.78	0.12	(2.55, 3.01)	
dictator fair (1/0)	−1.23	0.13	(−1.49, −0.98)	1.00
male (1/0)	−0.29	0.13	(−0.54, −0.03)	1.00
dictator higher status (1/0)	0.35	0.14	(0.08, 0.63)	1.00
failed comprehension (1/0)	1.64	0.26	(1.14, 2.15)	1.00
paranoia	0.42	0.12	(0.18, 0.66)	1.00
subjective social status	0.10	0.13	(−0.15, 0.36)	0.55
fairness × higher status	−0.16	0.25	(−0.66, 0.33)	0.45
higher status × paranoia	−0.06	0.16	(−0.36, 0.25)	0.24
dictator lower status (1/0)	0.03	0.09	(−0.15, 0.20)	0.21
age	0.01	0.05	(−0.09, 0.11)	0.14

**Table 2.** Variables affecting harmful intent attribution in the political ideology task. Harmful intent was coded as a five-level ordinal categorical variable and set as the response term in a clm [36]. Political affiliation refers to the subject's political affiliation (0–100, liberal–conservative).

parameter	estimate	unconditional s.e.	confidence interval	relative importance
intercept 1|2	−0.62	0.06	(0.50, 0.74)	
intercept 2|3	1.52	0.07	(1.37, 1.67)	
intercept 3|4	2.09	0.09	(1.92, 2.27)	
intercept 4|5	2.91	0.12	(2.68, 3.14)	
in-group partner (1/0)	−0.47	0.12	(−0.70, −0.24)	1.00
dictator fair (1/0)	−1.18	0.12	(−1.42, −0.95)	1.00
male (1/0)	−0.28	0.12	(−0.52, −0.05)	1.00
failed comprehension (1/0)	0.77	0.28	(0.21, 1.32)	1.00
paranoia	0.54	0.11	(0.31, 0.76)	1.00
political affiliation	0.09	0.12	(−0.15, 0.33)	0.64
in-group partner × paranoia	0.21	0.24	(−0.27, 0.68)	0.52
in-group partner × dictator fair × paranoia	−0.12	0.31	(−0.73, 0.48)	0.25

### 3.3. Self-interest attribution

Participants attributed more self-interest to unfair partners in both tasks (electronic supplementary material, tables S2 and S3), and we found no effect of paranoia on self-interest attribution in either task (figure 3; electronic supplementary material, tables S2 and S3), as predicted. Political conservatism positively affected the tendency to attribute self-interest to the partner (estimate: 0.26, CI: 0.02, 0.50), and female participants also attributed more self-interest to partners in both tasks (electronic supplementary material, tables S2 and S3). In the social status task, we found that participants attributed less self-interest to unfair dictators when those dictators were perceived as being lower status than the participants (electronic supplementary material, table S2). In the same task, there was also a positive effect of failing at least one comprehension question on tendency to attribute self-interest to the partner, though all results are qualitatively robust to the exclusion of failed comprehenders from analyses.

## 4. Discussion

Participants were exposed to social threat in two separate Dictator Game experiments. This task can identify paranoid attributions because the motives underpinning unfair dictator decisions are ambiguous and could feasibly reflect self-interest or (arguably less parsimoniously) malevolent intentions. Inferring that dictators are motivated by harmful intentions is a reasonable proxy for live paranoid thinking. Participants made stronger harmful intent attributions when playing against a higher status dictator, and when playing against an out-group dictator, compared to when they played against equal or lower status dictators, or against in-group dictators, respectively. Pre-existing paranoia strongly and positively predicted harmful intent attribution in all conditions but, counter to our predictions, did not result in exaggerated responses to social threat. Instead, paranoid people made stronger harmful intent attributions overall, but their responses to social threat increased at the same rate as less paranoid participants. This suggests that paranoia reflects a lower threshold for detecting social threat rather than an exaggerated responsivity to it. Furthermore, contextual social threat can induce live paranoid attributions, even in people who do not have high levels of pre-existing paranoia.

These results pertain to epidemiological studies where ethnic minority status is a risk factor for paranoia but increased density of same-ethnicity individuals can protect against this effect [10]. Ethnicity is perceived as one of the most salient markers of group affiliation and this protective ‘ethnic-density’ effect may be partly driven by lowered concerns about negative interactions with out-groups. We tested a key hypothesis deriving from these results: beliefs about perceived out-group members' hostile intentions mediate paranoid attributions. Here, we selected political affiliation as a marker of group identity precisely because it tends to be polarizing—and our data support the hypothesis. Ideally, future studies would challenge this hypothesis with other salient group affiliations.

Our study also raises questions about whether paranoia depends on qualitatively different social reasoning or instead reflects typical social information processing with a lower threshold for social threat detection. Some studies have reported a ‘dose effect’ of paranoia on cognitive biases (e.g. [43,44]), suggesting that harmful intent attributions should become increasingly out of proportion as pre-existing paranoia increases. However, our data imply that, in the face of social threat, the harmful intent attributions made by highly paranoid people scale in a similar way to those made by non-paranoid people. This implies that the key processes behind chronically high paranoia might be those that maintain high levels of social threat awareness rather than those that impair the interpretation of the magnitude of social risk. Indeed, maintaining factors, such as worry, negative self-beliefs, anomalous experiences and poor sleep, have been identified as key in previous paranoia research [45].

As expected, low subjective social status was a positive predictor of pre-existing paranoia. However, paranoid ideation was also higher among individuals who self-reported being at the top end of the social ladder. Bearing in mind that this analysis was unplanned and should therefore ideally be replicated before drawing firm conclusions, this result raises several possibilities. The first is that perceiving oneself as different, rather than being low status, is what promotes paranoid thinking. The second is that individuals at the extremes of the social status distribution are more likely to be fearful of danger from others. Those at the top of the hierarchy might be targeted by those below them, and those at the bottom fear those who might attempt to coerce or exploit them [46]. Existing studies suggest that increased threat perception is associated with low rather than high social status [47], though studies of non-human species have shown that social dominance can be linked to elevated stress [48] and that this is most likely when dominance hierarchies are unstable (see also [49]). Research on paranoia has not explored the individuals at society's highest social ranks, meaning that a potential ‘paranoia of the elite’ has yet to be investigated.

Political conservatism had a small positive effect on pre-existing paranoia. This supports previous research indicating that political conservatism might stem from increased threat-sensitivity [50], being associated, for example, with increased sensitivity to images of threatening faces [51].

We also replicated key results from Raihani & Bell [26], namely that pre-existing paranoia positively predicts harmful intent attributions but not attributions of self-interest in the Dictator Game; and also that pre-existing paranoia does not interact with dictator fairness to increase harmful intent attributions. Moreover, in the current study and previously, we found that participants made stronger harmful intent attributions towards selfish (compared to fair) dictators, providing a strong basis for the conclusions of both studies.

### 4.1. Limitations

The parameters of the social interaction were relatively constrained: information about group affiliation and social status was restricted to a simple declaration by the experimenters. Although this declaration reliably produced group and rank-related changes in harmful intent attributions, the context is considerably more limited than it would be in face-to-face interactions.

Amazon Mechanical Turk offers access to a diverse sample of participants that is more representative of the US general population than typical samples comprised of undergraduates [52], and produces results that are internally consistent (e.g. [26]) and equivalent to those obtained under laboratory conditions [52] or using benchmark national samples (e.g. [53]). Nevertheless, MTurk samples have reduced ethnic diversity than the general population [54], and we cannot generalize these findings from US respondents to other cultures. In terms of clinically relevant selection biases, MTurk participants report higher levels of social anxiety, although they are not more likely to report emotional dysregulation at clinically relevant levels than other general population samples [55].

In terms of clinical implications, it is unclear to what extent these results will generalize to patients with paranoid delusions. Independent studies have shown full taxometric continuity between subclinical and clinical paranoia [2,3], suggesting that it is reasonable to expect generalizability across the spectrum of severity. We do not know whether people with frank paranoid delusions participated in this study, but we suggest it is likely that at least some of the high paranoia sample may have beliefs that would be diagnosed as delusional. From the total sample, 7% had GPTS scores above the clinical mean of patients with paranoid delusions reported by Green *et al*. [29]. Furthermore, schizophrenia and paranoid delusions do not seem to be an impediment to Internet use [56,57]. However, it is also likely that there are selection biases that affect to what extent the clinical population participate in these online studies and these may have affected the results—we assume to under-represent the most severely affected here.

To conclude, our data suggest that contextual social threat increases live paranoid attributions during social interactions and that paranoia across the full spectrum could represent a lowered threshold for detecting social threat. By contrast, our data offer little support for the idea that paranoia involves a dysregulated reaction to the magnitude of social threat, indicating that paranoia represents an altered baseline for social threat perception in the context of intact social threat responsivity.

## Supplementary Material

Supplementary tables and experimental instructions

## Data accessibility

All data and code to reproduce analyses are available at https://osf.io/bzhx3/.
