# Supplementary material for: Experimentally induced social threat increases paranoid thinking
Source: R Soc Open Sci. 2018 Aug 1;5(8):180569. doi: 10.1098/rsos.180569 (PMC6124070; doi:10.1098/rsos.180569)
Supplement: Supplementary tables and experimental instructions [file rsos180569supp1.docx]

**Title:**

**Experimentally-Induced Social Threat Increases Paranoid Thinking**

**Author names and affiliations:**

Vanessa Saalfeld ^a*^, Zeina Ramadan^b*^, Vaughan Bell^b,c^, Nichola J. Raihani^a^

a. Department of Experimental Psychology, University College London.

b. Division of Psychiatry, University College London.

c. South London and Maudsley NHS Foundation Trust

* These authors are joint first authors and have contributed equally to the study

**Contents**

Statistical Methods (p.2)

Table S1: Variables affecting paranoia score (p. 2)

Table S2. Variables affecting attribution of self-interest in the social status task. (p. 4)

Table S3. Variables affecting attributions of self-interest in the political ideology task. (p. 5)

Table S4. Subject responses to the question of how confident they were that they were playing against a real person. (p. 6)

Table S5. Subject responses to the question of whether they had participated in tasks similar to this previously. (p. 6)

The Green et al. Paranoid Thought Scales (p. 7)

Game Instructions (p. 10)

References (p. 18)

**Statistical Method:**

To compare the relative effect sizes and confidence intervals associated with input variables in the models, we used multi-model selection with model averaging (described in detail in Burnham & Anderson 2002 and Grueber et al. 2011). This approach involves specifying a priori a candidate set of possible models and then comparing across all these models to see which model, or subset of models, is most consistent with the data. Models are compared using an AIC value (AICc, corrected for small sample sizes), which includes a penalty for including additional terms in the model. Lower AIC values indicate that the model is more consistent with the observed data. For each analysis, we first specified a global model containing all fixed effects and specified interactions. All possible models deriving from this global model are compared, resulting in a top model set containing all models that are within 2 AICc units of the ‘best’ model (that with the lowest AICc value). Parameter estimates are obtained by averaging across this top model set, thereby incorporating the uncertainty over the true parameter estimate when many models have similar levels of support. We report full model estimates and confidence intervals, which are conservative.

**Table S1. Variables affecting pre-existing paranoia score.**

| **Parameter** | **Estimate** | **Unconditional**  **SE** | **Confidence Interval** | **Relative Importance** |
| --- | --- | --- | --- | --- |
| *Intercept 1\|2* | *-1.76* | *0.06* | *(-1.88, -1.64)* |  |
| *Intercept 2\|3* | *-1.11* | *0.05* | *(-1.22, -1.01)* |  |
| *Intercept 3\|4* | *-0.61* | *0.05* | *(-0.70, -0.51)* |  |
| *Intercept 4\|5* | *-0.08* | *0.05* | *(-0.17, 0.01)* |  |
| *Intercept 5\|6* | *0.38* | *0.05* | *(0.29, 0.47)* |  |
| *Intercept 6\|7* | *0.89* | *0.05* | *(0.80, 0.99)* |  |
| *Intercept 7\|8* | *1.46* | *0.06* | *(1.35, 1.57)* |  |
| *Intercept 8\|9* | *2.30* | *0.07* | *(2.15, 2.44)* |  |
| SES High (1 / 0) | 1.31 | 0.33 | (0.67, 1.95) | 1.00 |
| Age | -1.14 | 0.08 | (-1.31, -0.98) | 1.00 |
| SES Ladder | -0.26 | 0.10 | (-0.45, -0.06) | 1.00 |
| Political Ideology | 0.18 | 0.08 | (0.03, 0.34) | 1.00 |
| SES Low (1 / 0) | 0.15 | 0.19 | (-0.23, 0.53) | 0.54 |
| Male (1 / 0) | 0.06 | 0.08 | (-0.10, 0.21) | 0.49 |

Paranoia was measured using the Green et al. Paranoid Thoughts Scale (2008). This table shows the full model-averaged estimates, unconditional standard-errors and confidence intervals associated with variables present in the top model set (where top models were all those within 2 AIC units of the best model). Relative importance is equivalent to the sum of the Akaike weights and indicates the probability that the term in question is a component of the best model.

Paranoia was converted into a nine-level, ordinal categorical variable and this was set as the dependent term in a cumulative link model (clm) using the package *ordinal* (Christensen 2015) in R version 3.4.1. All continuous input variables were standardized and binary input variables were centered (following Gelman 2008) so estimates can be considered to be on the same scale. ‘SES high’ is a binary dummy variable set as 1 if self-reported subjective social status was level 9 or 10 on the MacArthur ladder and 0 otherwise. ‘SES Low’ is a binary dummy variable set as 1 if self-reported subjective social status was 1 or 2 on the MacArthur ladder and 0 otherwise.

**Table S2. Variables affecting attribution of self-interest in the social status task.**

| **Parameter** | **Estimate** | **Unconditional**  **SE** | **Confidence Interval** | **Relative Importance** |
| --- | --- | --- | --- | --- |
| *Intercept 1\|2* | *-3.04* | *0.12* | *(-3.27, -2.80)* |  |
| *Intercept 2\|3* | *-1.98* | *0.11* | *(-2.19, -1.77)* |  |
| *Intercept 3\|4* | *-1.04* | *0.10* | *(-1.24, -0.84)* |  |
| *Intercept 4\|5* | *0.45* | *0.10* | *(0.25, 0.64)* |  |
| Dictator Fair (1 / 0) | -4.90 | 0.20 | (-5.29, -4.52) | 1.00 |
| Failed Comprehension (1 / 0) | 1.06 | 0.34 | (0.40, 1.72) | 1.00 |
| Male (1 / 0) | -0.28 | 0.13 | (-0.53, -0.03) | 1.00 |
| Lower status (1 / 0) | -0.38 | 0.15 | (-0.68, -0.08) | 1.00 |
| Dictator Fair:Low status | 0.79 | 0.29 | (0.22, 1.35) | 1.00 |
| Age | 0.01 | 0.06 | (-0.11, 0.13) | 0.18 |
| Paranoia | -0.01 | 0.06 | (-0.13, 0.11) | 0.18 |
| Higher status (1 / 0) | -0.03 | 0.09 | (-0.20, 0.15) | 0.21 |

‘Self-interest’ was coded as a five-level ordinal categorical variable and set as the response term in a clm. ‘Failed Comprehension’ was a binary dummy variable set as 1 if the participant answered at least one comprehension question incorrectly. ‘Lower status’ and ‘Higher status’ are two binary dummy variables, coded as 1 if participant had a lower or higher (respectively) self-reported subjective social status than the partner.

**Table S3. Variables affecting attributions of self-interest in the political ideology task.**

| **Parameter** | **Estimate** | **Unconditional**  **SE** | **Confidence Interval** | **Relative Importance** |
| --- | --- | --- | --- | --- |
| *Intercept 1\|2* | *-2.84* | *0.11* | *(-3.05, -2.63)* |  |
| *Intercept 2\|3* | *-1.74* | *0.09* | *(-1.93, -1.55)* |  |
| *Intercept 3\|4* | *-0.84* | *0.09* | *(-1.01, -0.66)* |  |
| *Intercept 4\|5* | *0.49* | *0.09* | *(0.31, 0.66)* |  |
| Dictator Fair (1 / 0) | -4.45 | 0.17 | (-4.79, -4.11) | 1.00 |
| Male (1 / 0) | -0.30 | 0.12 | (-0.54, -0.06) | 1.00 |
| Conservatism | 0.26 | 0.12 | (0.02, 0.50) | 1.00 |
| Age | -0.24 | 0.12 | (-0.48, 0.00) | 1.00 |
| In-group Partner (1 / 0) | 0.12 | 0.13 | (-0.13, 0.37) | 0.65 |
| Paranoia | 0.05 | 0.10 | (-0.15, 0.24) | 0.41 |
| In-group Partner x Paranoia | -0.06 | 0.16 | (-0.38, 0.26) | 0.15 |
| Failed Comprehension (1 / 0) | -0.01 | 0.10 | (-0.21, 0.19) | 0.10 |

‘Self-interest’ was coded as a five-level ordinal categorical variable and set as the response term in a clm. Conservatism is the extent to which participants rated themselves as supporting conservative political parties on a scale of 0-100, where 0 = liberal and 100=conservative. ‘In-group partner’ denotes whether the partner was politically affiliated (1) or counter-affiliated (0) to the participant..

**Table S4.** Subject responses to the question of how confident they were that they were playing against a real person.

| **Task** | **1**  **(not at all confident)** | **2** | **3** | **4** | **5**  **(extremely confident)** | **N** |
| --- | --- | --- | --- | --- | --- | --- |
| **Social Status** | 168 | 184 | 235 | 328 | 327 | 1,242 |
|  | 13.5 % | 14.8 % | 18.9 % | 25.1 % | 26.3 % |  |
| **Political Ideology** | 187 | 202 | 253 | 338 | 328 | 1,308 |
|  | 14.3 % | 15.4 % | 19.3 % | 25.8 % | 25.1 % |  |

**Table S5.** Subject responses to the question of whether they had participated in tasks similar to this previously.

| **Task** | **1**  **(nothing like this)** | **2** | **3** | **4** | **5**  **(exactly like this)** | **N** |
| --- | --- | --- | --- | --- | --- | --- |
| **Social Status** | 177 | 206 | 364 | 382 | 113 | 1,242 |
|  | 14.3 % | 16.6 % | 29.3 % | 30.8 % | 9.10 % |  |
| **Political Ideology** | 212 | 216 | 371 | 386 | 123 | 1,308 |
|  | 16.2 % | 16.5 % | 28.4 % | 29.5 % | 9.40 % |  |

We note one additional unexplored analysis in this section, which we ran to check whether participant scepticism that the partner was real affected the tendency to attribute harmful intentions to the partner. Including ‘skepticism’ as a term in the models reported in the main text does not qualitatively change the results.

**The Green et al. (2008) Paranoid Thoughts Scale**

To begin, **please enter your Amazon Mechanical Turk WorkerID** here:

(Please see below for where you can find your Worker ID.)

Your Worker ID starts with the letter A and has 12-14  letters or numbers. It is NOT your email address. If we do not have your correct Worker ID we will not be able to pay you.

Your WorkerID can be found on your dashboard page.

You are about to take part in an academic study which is run by the Raihani Lab, based at University College London. This project has been approved by the UCL Ethics Board project 3720/001.

By continuing, you are consenting to allow the Raihani Lab to use your responses in the study for academic purposes.

The purpose of this study is to understand people's behaviour. All data are anonymous (your name will not appear in any publication related to this study and will not be shared with any other parties). By completing this HIT you will be granted a qualification to participate in a subsequent HIT run by Raihani Lab in the future. You will be notified when the next HIT becomes available.

Please tick 'I agree' if you agree to these conditions. If you do not wish to participate, or if you change your mind during the course of the study, please close this window.

- I agree (1)

Please read each of the following statements carefully. They refer to thoughts and feelings you may have had about others **over the last month**. Think about last month and indicate the extent of these feelings from **1 (Not at all) to 5 (Totally).**

**Please complete both Part A and Part B.**

(N.B. Please do not rate items according to any experiences you may have had under the influence of drugs.)

**Part A:**

|  | Not at all (1) | 2 | 3 | 4 | Totally (5) |
| --- | --- | --- | --- | --- | --- |
| 1. I spent time thinking about friends gossiping about me. |  |  |  |  |  |
| 2. I often heard people referring to me. |  |  |  |  |  |
| 3. I have been upset by friends and colleagues judging me critically. |  |  |  |  |  |
| 4. People definitely laughed at me behind my back. |  |  |  |  |  |
| 5. I have been thinking a lot about people avoiding me. |  |  |  |  |  |
| 6. People have been dropping hints for me. |  |  |  |  |  |
| 7. I believed that certain people were not what they seemed. |  |  |  |  |  |
| 8. People talking about me behind my back upset me. |  |  |  |  |  |
| 9. I was convinced that people were singling me out. |  |  |  |  |  |
| 10. I was certain that people have followed me. |  |  |  |  |  |
| 11. Certain people were hostile towards me personally. |  |  |  |  |  |
| 12. People have been checking up on me. |  |  |  |  |  |
| 13. I was stressed out by people watching me. |  |  |  |  |  |
| 14. I was frustrated by people laughing at me. |  |  |  |  |  |
| 15. I was worried by people's undue interest in me. |  |  |  |  |  |
| 16. It was hard to stop thinking about people talking about me behind my back. |  |  |  |  |  |

**Part B:**

|  | Not at all (1) | 2 | 3 | 4 | Totally (5) |
| --- | --- | --- | --- | --- | --- |
| 1. Certain individuals have had it in for me. |  |  |  |  |  |
| 2. I have definitely been persecuted. |  |  |  |  |  |
| 3. People have intended me harm. |  |  |  |  |  |
| 4. People wanted me to feel threatened, so they stared at me. |  |  |  |  |  |
| 5. I was certain people did things in order to annoy me. |  |  |  |  |  |
| 6. I was convinced there was a conspiracy against me. |  |  |  |  |  |
| 7. I was sure someone wanted to hurt me. |  |  |  |  |  |
| 8. I was distressed by people wanting to harm me in some way. |  |  |  |  |  |
| 9.I was preoccupied with thoughts of people trying to upset me deliberately. |  |  |  |  |  |
| 10. I couldn't stop thinking about people wanting to confuse me. |  |  |  |  |  |
| 11. I was distressed by being persecuted. |  |  |  |  |  |
| 12. I was annoyed because others wanted to deliberately upset me. |  |  |  |  |  |
| 13. The thought that people were persecuting me played on my mind. |  |  |  |  |  |
| 14. It was difficult to stop thinking about people wanting to make me feel bad. |  |  |  |  |  |
| 15. People have been hostile towards me on purpose. |  |  |  |  |  |
| 16. I was angry that someone wanted to hurt me. |  |  |  |  |  |

**Game Instructions**

**Where instructions and/or information was the same across both tasks, we do not duplicate instructions.**

*General Consent Screen (same in both tasks)*

You are about to take part in an academic study, which is run by the Raihani Lab, based at University College London. This project has been approved by the UCL Ethics Board project 3720/001.

**Please note that the Raihani Lab does not use deception. All participants are real.**

By continuing, you are consenting to allow the Raihani Lab to use your responses in the study for academic purposes.   The purpose of this study is to understand people's behaviour. All data are anonymous (your name will not appear in any publication related to this study and will not be shared with any other parties).

Please tick 'I agree' if you agree to these conditions. If you do not wish to participate, or if you change your mind during the course of the study, please close this window.

- I agree (1)

***Social Status Task (example shown where participant LOWER status than partner)***

**Task A**
 You are Player 2.
 You have been allocated a bonus of **$0.00**.

 Player 1 is your partner in this task.
 Player 1 has been allocated a bonus of **$0.50**.

 Player 1 could choose one of the following options:
   keep $0.50 and send $0.00 to you keep $0.25 and send $0.25 to you
 Player 1 has already made their decision.

Previously, you saw the ladder below representing people in your country from being worst off (1) to being best off (10).

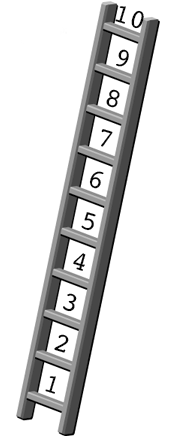


You chose a number between 1 and 10 that best represented where you currently stand in your life compared to other people in your country.

Player 1  - your partner in this task - rated themselves as being **below you on this ladder.**

First, we would like you to answer a few questions to ensure you have understood the game.

 Please note that you will only be allowed to proceed in this task and receive your bonus if you answer these questions correctly.

***Comprehension Questions (Comp Q1 and Q2 same in both tasks)***

Comp Qu 1. What was Player 1's starting bonus? (correct answer in bold)

- $10 (1)
- $5 (2)
- **$0.50 (3)**

Comp Qu 2. If Player 1 sent $0.25 to you, how much would they keep for themselves?

- $0.00 (1)
- $0.50 (2)
- $**0.25** (3)

Comp Qu 3. Is Player 1 higher, lower or on the same rung of the social ladder as you?

- **Player 1 reported being higher than me (1)**
- Player 1 reported being below me (2)
- Player 1 said they were on the same rung as me (3)

*Dictator Decisions (same in both tasks)*

*Fairness Condition: Unfair*

Player 1 decided to keep $0.50 and send $0.00 to you.

 That means Player 1 gets $0.50 and you get $0.00.

*Fairness Condition: Fair*

Player 1 decided to keep $0.25 and send $0.25 to you.

 That means Player 1 gets $0.25 and you get $0.25.

*Intention Attribution (same in both tasks)*

Please use the slider below to indicate the extent to which you believe Player 1's decision was driven **by their desire to earn money** in the game.

| 1 (1) | 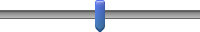 |
| --- | --- |

Please use the slider below to indicate to what extent Player 1's decision was driven **by their desire to reduce your bonus** in the game.

| 1 (1) | 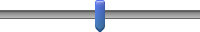 |
| --- | --- |

***Participant in Dictator Role***

***This example shows condition where participant was informed that partner was above them on the social ladder. These data were not analysed.***

**Task B**

 Now, you are Player 1.
 You have been allocated a bonus of **$0.50**.

 Your partner is Player 2.
 Player 2 has been allocated a bonus of **$0.00**.   Your worker ID will not be revealed to the other player and you will not find out their worker ID.  
 Your partner has indicated that they are ABOVE you on the social ladder.
  
You can choose to **send none or half** of your bonus to Player 2.

 For example: If you choose to send $0.00, then you will keep $0.50 and Player 2 will get $0.00. If you choose to send $0.25, then you will keep $0.25 and Player 2 will get $0.25.

Please indicate your decision below.

- I want to keep $0.50 and send $0.00 to Player 2 (1)
- I want to keep $0.25 and send $0.25 to Player 2 (2)

*Demographic and other questions (same in both tasks)*

Thanks - it's nearly the end! Before you go, it would be very helpful if you could answer some additional questions to help with our research.

Unlike some other research labs, Raihani Lab **does not** use deception on MTurk. All participants are real. Nevertheless, for our own purposes, it is helpful to know to what extent you believed that the other player really existed.

|  | 1 - Very skeptical that other player was real (1) | 2 (2) | 3 (3) | 4 (4) | 5 -Very confident that other player was real (5) |
| --- | --- | --- | --- | --- | --- |
| Please choose one (1) |  |  |  |  |  |

To what extent have you participated in HITs similar to this before?

|  | 1 - Nothing like this scenario (1) | 2 (2) | 3 (3) | 4 (4) | 5 - Exactly like this scenario (5) |
| --- | --- | --- | --- | --- | --- |
| Please choose one (1) |  |  |  |  |  |

What is your age?

________________________________________________________________

What is your gender

- Male (1)
- Female (2)

***Political Ideology Task***

***Example where partner is in-group member.***

**Task A**

You are Player 2.
 You have been allocated a bonus of **$0.00**.

 Player 1 is your partner in this task.
 Player 1 was allocated a bonus of **$0.50**.

 Player 1 could choose one of the following options:
   keep $0.50 and send $0.00 to you keep $0.25 and send $0.25 to you
 Player 1 has already made their decision.

You previously indicated your political orientation on the following slider scale.


    
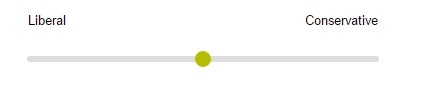


 Player 1 has **similar** political views to you and they were informed of this before making their decision.

**References**

Christensen RH. Package ‘ordinal’. <https://cran.r-project.org/web/packages/ordinal/index.html> 2015.

Gelman A. Scaling regression inputs by dividing by two standard deviations. Statistics in Medicine. John Wiley & Sons, Ltd; 2008 Jul 10;27(15):2865–73.

Green CEL, Freeman D, Kuipers E, Bebbington P, Fowler D, Dunn G, et al. Measuring ideas of persecution and social reference: the Green et al. Paranoid Thought Scales (GPTS). Psychological Medicine. 2007;38(01).
